# Association between Lateral Cephalometric Changes in X-Y Coordinate System and Profile Changes among Skeletal Class III Patients after Orthognathic Surgery

**DOI:** 10.29252/wjps.9.3.282

**Published:** 2020-09

**Authors:** Massoud Seifi, Mahsa Jafarpour Boroujeni, Reza Tabrizi, Soodeh Tahmasbi

**Affiliations:** 1 Department of Orthodontics, Dental School, Shahid Beheshti University of Medical Sciences, Tehran, Iran;; 2 Department of Oral and Maxillofacial Surgery, Dental School, Shahid Beheshti University of Medical Sciences, Tehran, Iran

**Keywords:** Cephalometric analysis, Orthognathic surgery, Skeletal class III, X-Y coordinate system

## Abstract

**BACKGROUND:**

Since aesthetic issues are the most important causes of referring skeletal class III patients to surgeons, investigating the impact of orthognathic surgeries on improving patient profiles increases the quality of treatment and quality of life.

**METHODS:**

In a retrospective observational-analytical study, 25 patients older than 18 years with class III skeletal malocclusion who had gone under both orthodontic and double-jaw orthognathic treatment were enrolled. Cephalometric imaging interval was before and at least 6 months after surgery. By defining a number of points and coordinate axes (X-Y), a criterion for comparing hard and soft tissue changes was obtained. These measurements were coordinated, linear and angular. The quantitative data were compared with data obtained using the Likert Scale Questionnaire by means of electronic “Google Forms” that was completed by orthodontists (n=5) and maxillofacial surgeons (n=5) to rank improvement in post-surgical profiles for both cephalometry and photography from poor to pleasant. Spearman Correlation Analysis was conducted between the quantitative and qualitative data.

**RESULTS:**

Vertical changes of point B and horizontal changes of point PNS showed correlation with improvement of patient profile. Changes in N-Pog line (R=-0.4), mandibular plane angle (R=-0.4) and nasolabial angle (NLA) (R=0.38) were significantly correlated with improvement of profiles.

**CONCLUSION:**

In orthognathic double-jaw surgery on patients with skeletal Class III, forward movement of maxilla, upward positioning of mandible (decreasing anterior facial height), decreasing mandibular plane angle and increasing nasolabial angle would result in a better profile.

## INTRODUCTION

All humans like beauty and interpret it as a relative concept with various definitions. Predicting patient profile after orthognathic surgery and achieving maximal beauty has always been one of the primary goals of maxillofacial surgeons. Surgeons and orthodontists carefully plan for each patient to get the best results with the least complications. However, it should be noted that complications are part of treatments. Preoperative imaging and analysis of the impact of points and angles on function and beauty would identify root causes of the problems to the specialists, so that the treatment team can provide the best treatment.^1^

Since the most important cause of skeletal class III patients referring to maxillofacial surgeons is aesthetics, investigating the effect of changes made during orthognathic surgeries on aesthetics and improving patient profiles would improve the predictability of treatment outcome. In order to improve the treatment of orthognathic patients, it is necessary to carefully consider points such as diagnosis, treatment plan, biomechanical principles and timing of treatment. With the advent of diagnosis and treatment in the nineteenth century and reaching its peak in 1980 and improving until the beginning of the year 2000, significant progress has been made in this area.^1^

With the development of antibiotics and advances in surgical and rigidfixation techniques, orthognathic therapies have become more practical. Using the new techniques and orthodontists can make predictions about the timing and types of surgical techniques. Ultimately, development of these techniques has made orthognathic therapies to become predictable treatments with remarkable results in facial beauty and function.^1^ In recent years, introduction of CBCT and CASS devices has greatly assisted orthodontists, maxillofacial surgeons and patients, and has pioneered new approaches. Both of these techniques have helped the treatment team to understand the three-dimensional nature of the abnormalities and establish an appropriate treatment plan. Although planning is very important in treatment, the added advantage of 3D images is that it allows the specialist to perform the failure analysis, which is essentially the same trial and error.^2^

Numerous studies have shown that two-dimensional radiographs to be obtained from CBCT scans. Two-dimensional images of 3D structures can accelerate the diagnosis and cephalometric analysis is one of the important steps in the diagnosis and treatment of patients with acute skeletal malocclusion. There are numerous analyzes that each orthodontist or surgeon uses individually, while the most common orthodontic analyse uses angles that are suitable for assessing the position of the tooth, but are much less accurate in evaluating the position of the jaw. Although new 3D imaging techniques such as mirroring allow the specialist to simulate exactly what he or she intends to do in practice, linear-angular data analysis is easier and more understandable in two-dimensional imaging, and the patient is exposed to less radiation. So final decision of the surgeons would be based on the patient’s soft tissue, and 2D imaging analysis that would be more safe and functional.^3^

In this study, a coordinate plane was designed in two dimensions regarding lateral cephalometry before and after surgery of skeletal class III patients. Profile improvement index and rate of change in all three types of coordinates, linear and angular variables before and after surgery and their relationship were investigated to identify the most determinant factors in improving the patient profile. The knowledge gained from this research can have a significant impact on the diagnosis, treatment plan and considerations of orthognathic surgery.

## MATERIALS AND METHODS

The present study was a retrospective observational-analytical one, and the data collection method was a qualitative questionnaire, and the measurement tables were completed using lateral cephalometric radiographs. All patient rights were reserved. Twenty-five Class III skeletal patients with mean age of 23 years old who underwent orthognathic surgery were selected from the archives of Shahid Beheshti Dental School. Only four patients received advancement genioplastic surgery too. 

Cephalometric radiographs were provided at two time points, before orthodontic initiation (T1) and after surgery following removal of fixed orthodontic device (at least 6 months after surgery) (T2) and under standard conditions, head in the state of NHP, habitual occlusion status and the relaxation state of the lips. In this study, numerical values of variables before (T1) and after (T2) surgery were considered as quantitative variables. These variables fall into three categories of coordinate, linear and angular. A Likert Qualitative Questionnaire was also given to 10 orthodontists and maxillofacial surgeons to evaluate the improvement of profiles (photography and radiography) of the patients and by calculating the mean of these qualitative numbers for each sample, the patient profile score which was regarded as the qualitative variable of this study was obtained.

**Fig. 1:**
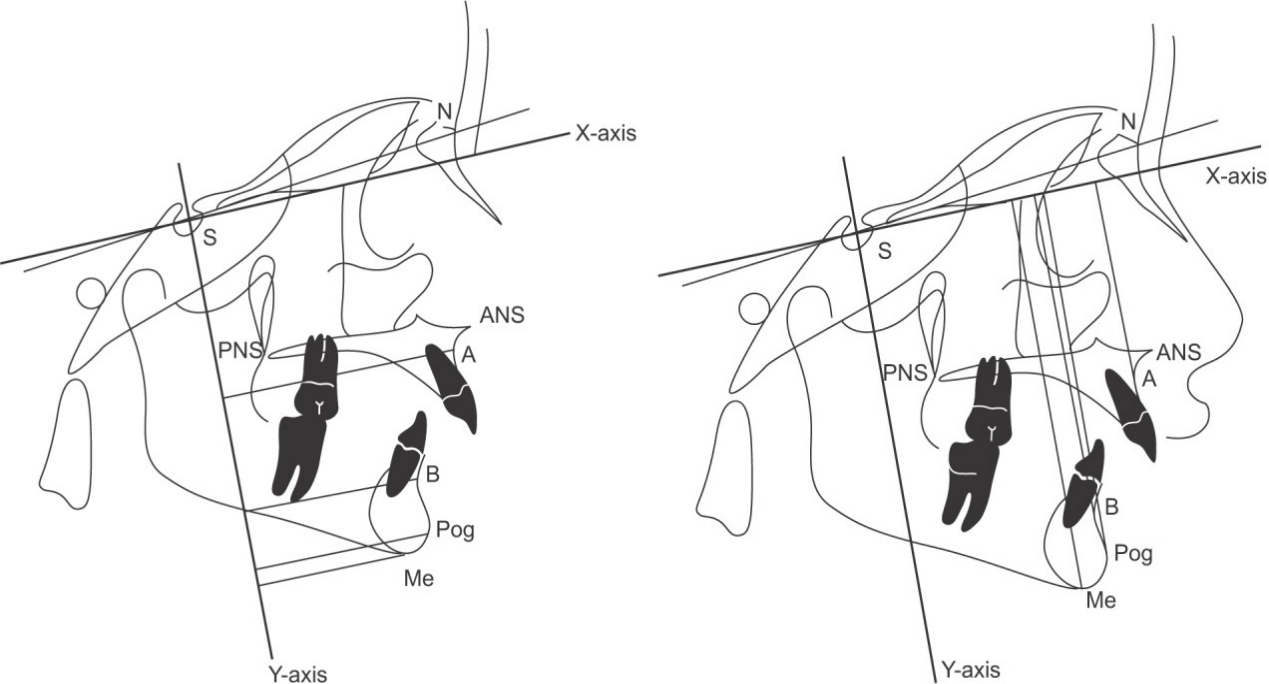
X-Y coordinate axis

To evaluate displacement of soft and hard tissue points in two dimensions, an X-Y coordinate plane has been designed. In each sample, the lateral cephalometry of the X-axis was drawn at a 7-degree angle below the SN line in a way that the Y-axis would be perpendicular to the X-axis.^4^ Accordingly, each defined anatomical point would have two variables of X and Y, while the magnitude of X is equal to the size of the perpendicular to the Y-axis and the magnitude of Y is equal to the size of the perpendicular to the X-axis (Figure 1). In this study, displacement of defined points in the patient’s lateral cephalometry before and after treatment was obtained based on X and Y indices in this coordinate plane. In addition to defining the points and examining their displacement in two dimensions, the linear and angular coordinate variables were also examined.

**Fig. 2:**
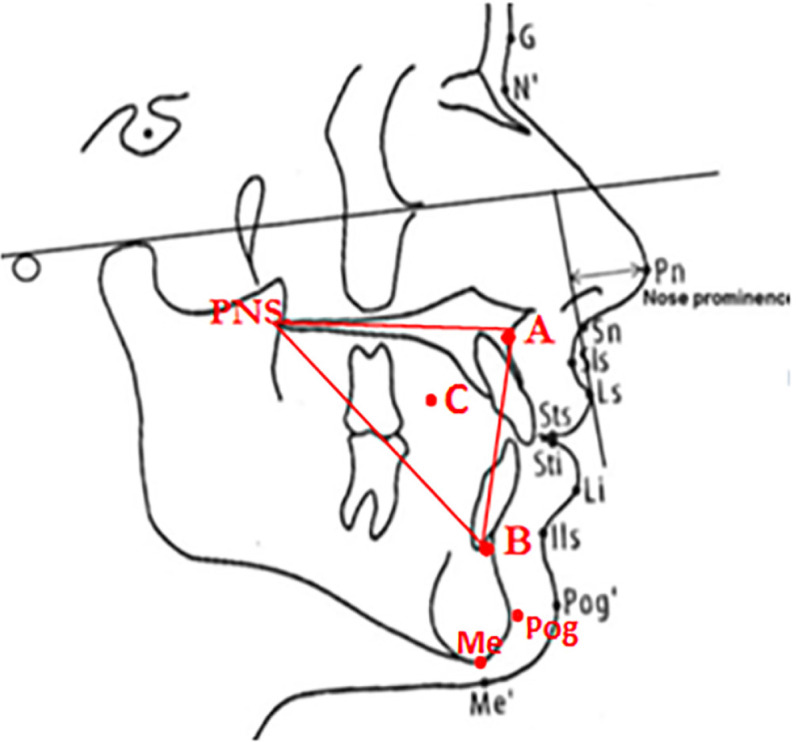
Soft and hard tissue

**Fig. 3:**
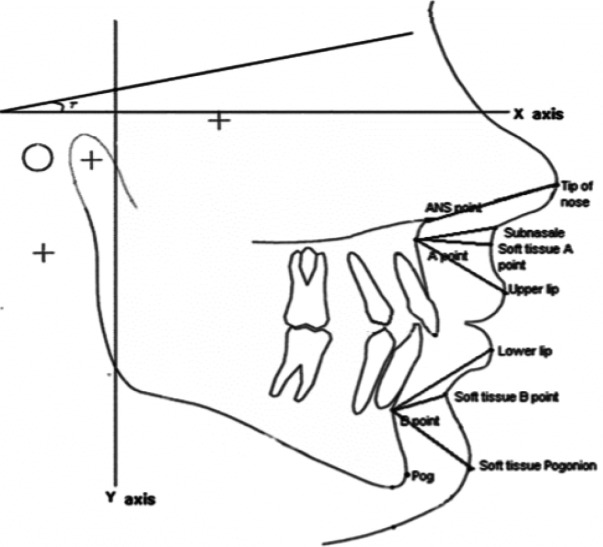
Soft tissue thickness in upper lip area

**Fig. 4:**
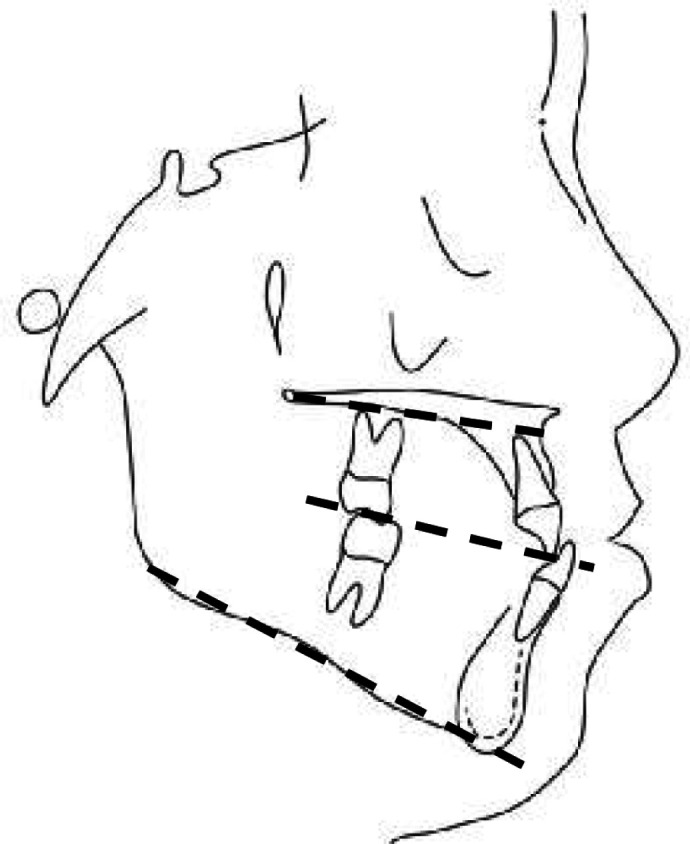
Maxillary, occlusal and mandibular planes

**Fig. 5:**
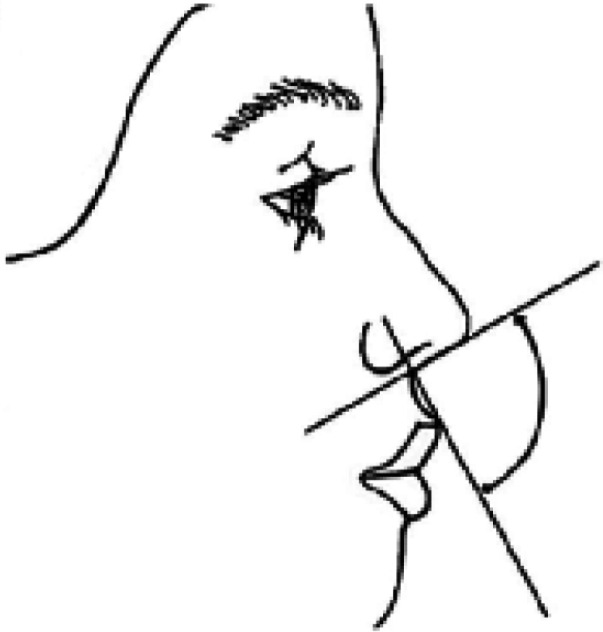
NLA Angle Drawing.^7^

Soft tissue indices in lateral cephalometry were selected according to the research by Isiekwe *et al.* (G point was eliminated and C point the center of Triangle (A, B, PNS) was added) (Figure 2).^5^ Upper lip length and thickness, linear size of N-Pog and Sn were calculated from the patient’s lateral cephalometry before and after treatment. The upper lip length was the distance between Sn to Ls. The mean upper lip thickness in these three regions (A-Sn, A-Sls, A-Ls) was considered as the variable of the soft tissue thickness (Figure 3).^6^ Maxillary, Functional Occlusal and Mandibular plane angles to Sn line (Figure 4) and NLA angle (Figure 5) were considered as angular variables.^7^

**Fig. 6:**
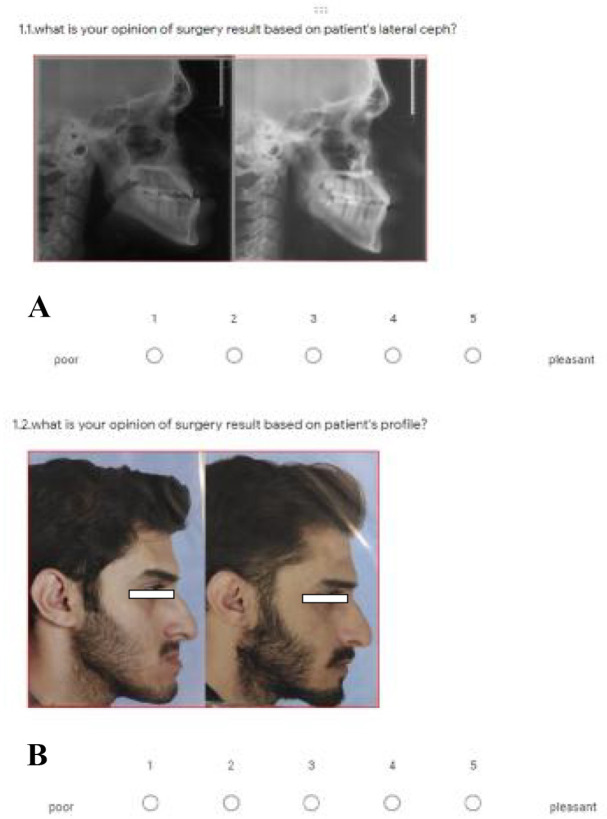
Profile of improvement assessment questionnaire in the format of Google Form. **A.** Hard tissue profile, **B.** Soft tissue profile

All of the coordinate, linear and angular variables in the form of T2-T1 were finally statistically analyzed. Furthermore, to obtain the range of subsequent orthogonal change points on the X-Y coordinate plane, as in the envelope of discrepancy, the two-dimensional point change range was displayed as a diagram (envelope of change). For reliability check, all measurements were performed on 12 samples at 20 days intervals by a senior dental student who had received the required training. A qualitative online profile questionnaire (in 50 questions) in Google Form format including pre- and post-treatment photography (soft tissue) and lateral radiography (hard tissue) for each patient was presented to five orthodontists and five maxillofacial surgeons to score from 1 to 5 (poor to pleasant). Then, the mean score of profile improvement was calculated for each patient (Figure 6).

In this study, after entering data into SPSS software (version 21, Chicago, IL, USA), the score of improvement of patient profiles was calculated as mean and for each defined point, angle and side, the values measured in the XY system at time T1 were subtracted from the values at T2, and were considered as quantitative variables for 25 samples. Spearman Correlation analysis was used to compare the relationship of points displacements with the patients’ profile score and P value <0.05 was considered to be significant. Correlation of soft and hard tissue alterations in three areas was also measured by Spearman Correlation. Partial correlation analysis was used to evaluate the influence of soft tissue thickness on hard and soft tissue displacement, and the upper lip soft tissue thickness variable was considered as the control variable. Given that the present study was retrospective, we did not intervene the patients and only the patients’ personal information and their identities were considered.

## RESULTS

In this study, 25 patients (16 females and 9 males) with Class III skeletal malformations with mean age of 23.1 years (18-31) were evaluated. These patients underwent double-jaw surgery. The variables of our study were classified into quantitative and qualitative groups. In the category of quantitative variables, three groups of variables fell into coordinate, linear, and angular points, while each was calculated before and after treatment. In the qualitative category, there was profile variable, which was the mean of scores that evaluators gave to soft and hard tissue profile improvement of patients.

For all cases, the sagittal and vertical indices for pre- and post-surgical treatment were traced (Table 1). Unlike vertical class III indices, horizontal ones changed significantly. However, in patients with good profile (>4), Jaraback index and mandibular plane angle changed significantly. Sagittal indices were ANB angle, SNB angle, SNA angle, and Wits analysis. Vertical indices were mandibular plane angle, occlusal angle, maxillary plane angle, and Jaraback index.

**Table 1:** Vertical and horizontal indices

**Cases**	**Profile score**	**T1**	**T2**	**T1**	**T2**	**T1**	**T2**	**T1**	**T2**	**T1**	**T2**		**T1**	**T2**		**T1**	**T2**	**T1**	**T2**
		**Jaraback index** (S-Go/N-Me)*100		**Wits** **(mm)**		**Maxillary plane angle**		**Occlusal plane angle**		**Mandibular plane angle**			**SNA angle**			**SNB angle**		**ANB angle**	
1	4.1	66.4	66.0	-11.4	-5.5	12	10	11	11	33		33	85		88	90	88	-5	0
2	4.5	55.5	54.4	-12.5	-3	20	17	19	18	44		45	88		82	93	79	-5	3
3	4.5	60.7	61.8	-10.5	-5	22	22	18	18	35		35	74		79	82	80	-8	-1
4	3.3	51.1	58.3	-8.5	0	18	21	21	22	48		51	78		82	80	78	-2	4
5	3.7	60.6	67.6	-4.5	4	18	20	18	15	42		46	75		80	76	75	-1	5
6	4.3	65.6	68.9	-11	-3.7	14	10	15	14	32		29	84		92	88	88	-4	4
7	3.8	63.2	63.2	-8.8	-4.4	24	22	15	17	40		38	75		76	82	79	-7	-3
8	4	59.2	64.6	-10	-4.	14	13	13	8	35		31	83		85	88	86	-5	-1
9	4.2	57.3	60.5	-18	-8	20	17	21	20	43		37	79		85	86	85	-7	0
10	3	59.2	56.2	-15	-5	23	22	17	27	47		48	81		89	83	83	-2	6
11	3.5	62.1	61.2	-12.3	-6.5	13	18	11	13	34		35	80		86	88	86	-8	0
12	3.4	58.2	57.7	-19	-12	20	23	20	20	40		38	79		77	88	83	-9	-6
13	4.3	61.5	60.1	-16	-7.5	25	15	18	14	42		40	77		83	84	86	-7	-3
14	3.9	63.8	65.2	-12	-6.5	15	10	9	12	28		30	80		85	89	87	-9	-2
15	4	55.8	61.3	-12	-9.5	15	23	21	25	44		40	79		79	84	79	-5	0
16	1.8	6	52.8	-6	3.5	21	21	22	25	43		49	78		81	81	73	-3	8
17	2.7	61.9	60.8	-14	-4.5	27	28	20	21	42		44	79		85	83	82	-4	3
18	3.6	67.2	69.6	-13.5	-5.5	20	24	19	14	32		30	76		82	84	84	-8	-2
19	3.5	64.5	68.8	-9	-6.5	19	20	12	16	31		30	79		84	85	86	-6	-2
20	3.8	68.7	67.1	-6.5	-1.5	15	15	14	11	30		30	73		84	76	83	-3	1
21	2.8	75.4	71.4	-17	-7.5	10	10	6	4	19		21	87		87	96	92	-9	-5
22	4.1	68.8	71.9	-11	-5	13	16	12	16	29		28	80		83	86	83	-6	0
23	4.1	60	63.8	-5	-2.5	18	20	15	17	40		33	83		79	83	79	0	0
24	3.7	60.5	59.8	-18	-5	18	20	22	20	42		38	76		80	81	81	-5	-1
25	3.3	64.7	62.9	-9	-5	18	16	15	20	35		30	80		82	83	81	-3	1

**Fig. 7:**
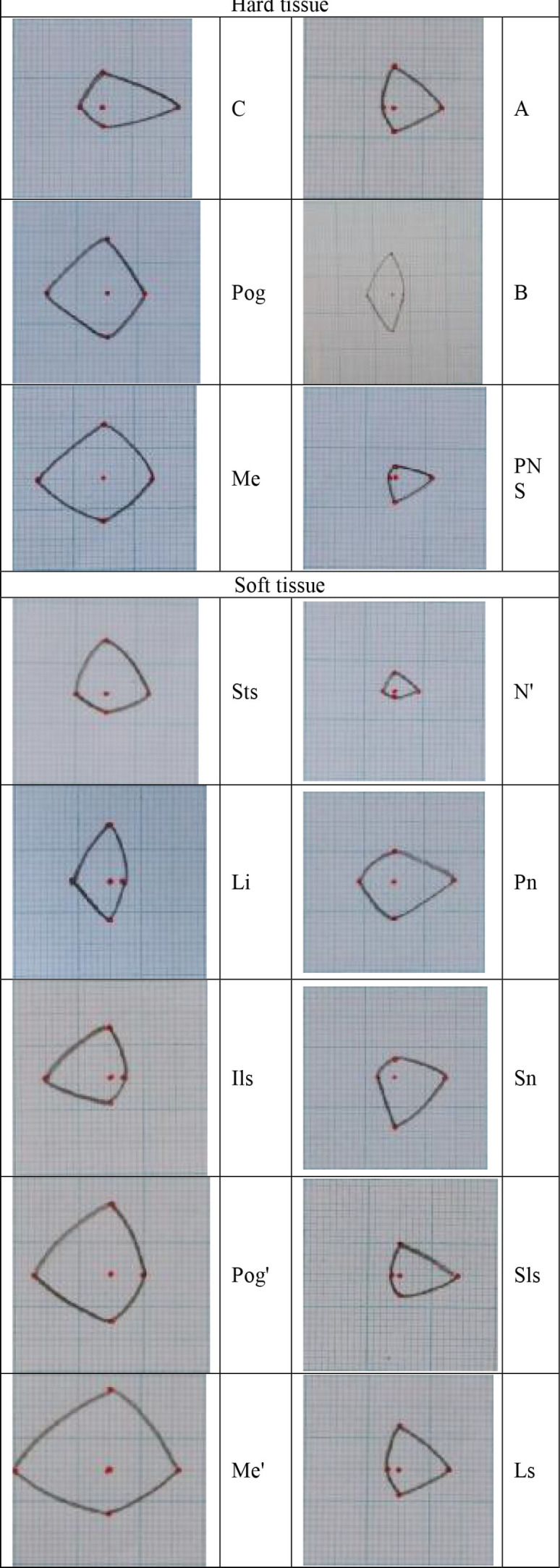
Landmarks’ envelope of changes (images have the same scale and are comparable).

The highest numerical value of the envelope of change was calculated for the horizontal displacement of point Me’ (-15.4 mm), vertical displacement of point Me’ (+13.9 mm) and horizontal displacement of point C (+13.5 mm). The lowest numerical value of the envelope had three points of A, Sls and PNS (-1.4 mm) and vertical displacement of point N’ (-1 mm). The highest mean of displacement changes were calculated for the horizontal displacements of three points Ils (-3.7), A (+3.3 mm) and Sls (+3.1 mm) (Figure 7).

According to the Spearman Correlation test, vertical (Y) and horizontal (X) displacements of all points with profile score of the patients were not significant (*p*>0.05), except for horizontal displacement (X) of PNS point (R=-0.38) and vertical displacement (Y) of point B (R=-0.44). In the correlational study of the profile of patients with changes in linear and angular variables, N-Pog line (R=-0.4), mandibular plane angle (R=-0.4) and NLA angle (R=0.38) were significant (*p*<0.05). In cases with good profile score (>4), the correlation between profile score and horizontal displacement of point A (R=+0.65), PNS(R=-0.78) and upper lip length were significant. 

According to Spearman Correlation analysis, vertical and horizontal displacement of point A and horizontal displacement of point B were not significantly correlated with occlusal plane angular changes (*p*>0.05). Only vertical displacement of point B was significantly correlated with occlusal plane angular changes (R=0.4). According to Spearman’s analysis, the NLA angle variations did not show significant correlation with maxilla, mandibular and occlusal planes angles (*p*>0.05). 

Spearman analysis was also used to examine the correlation between hard tissue and beneath soft tissue in three areas. Results showed that for the points (A and Sls), (B and Ils), (Pog and Pog’), horizontal and vertical displacement were statistically significant (*p*<0.05). Correlation coefficients for the horizontal and vertical displacement were shown separately in Table 2. The horizontal displacement correlation was higher for all three points in comparison to the vertical ones. Among these three areas for both vertical and horizontal dimensions, chin prominence showed the highest correlation and the upper lip revealed the least. 

**Table 2:** Correlation coefficient of hard and soft tissue displacement

**Landmarks**	**Vertical displacement**	**Horizontal displacement**
	**Correlation coefficient (R)**	**Correlation coefficient (R)**
A, Sls	0.49	0.6
B and Ils	0.5	0.7
Pog and Pog’	0.69	0.8

In 23 patients, the upper lip length increased after treatment, and in only 2 patients, it decreased to approximately 1 mm. In general, we observed an increase in upper lip length by an average of 1.3±1.5 mm. The upper lip thickness of the patient was measured in three areas. In 14 cases, there was a decrease in lip thickness, while in 11 cases, it increased and the total mean was -0.4±1.5 mm. Assessing effect of soft tissue thickness, horizontal and vertical displacements of the corresponding hard and soft tissues, i.e. the points (A and Sls), (B and Ils), (Pog, and analyzed by Partial Correlation and; soft tissue thickness before treatment (to eliminate the surgical edema) was considered as the control variable. The correlation between the horizontal and vertical displacement of the corresponding points did not change significantly.

## DISCUSSION

One of the major treatment options for skeletal class III at post-puberty ages is orthognathic surgery. In order to help surgeons and orthodontists obtain the best profile outcome, the most important and determinative landmarks must be investigated. According to previous studies, the lower lip and chin are considered as the most important profile predictors. In this study, we found that sagittal indices significantly changed during surgery when compared with vertical ones. However Jaraback index and mandibular plane angle in vertical category changed significantly in pleasant profile cases.

In the Espinar study (class II patients), only horizontal displacements of points Ils and Pog’ had the most effect on the profile.^8^ In the present study, we observed that the higher the B point, the lower the PNS point, and the more the anterior point A, the better the profile would be. In the study of Marchiori *et al.*, participants’ profile was not correlated with magnitude of NLA angle.^9^ However, in our study, better profiles would be expected by decreasing nasolabial angle, anterior facial height and angle of mandibular plane. In Espinar *et al.*’s research (Class II patients), the highest displacements were in the lower third of the face; and Ils, Li, and Pog’ points showed the most significant changes (horizontal dimension).^8^

In a study by Altung Atac *et al.* (double-jaw surgery for Class III patients), the most changes were in the horizontal and vertical dimensions in the mentolabial angle and the chin. On the other hand, nose and upper lip areas had the least changes in these surgeries and questioned the necessity of maxillary surgery in the patients.^10^ In our study among pre- and post-surgical measurements, vertical movements of the upper lip and the nose were minimum in contrast with the vertical chin movement that was significant.

Besides horizontal movement of points A and Ils that were maximum in means, the Sls, Ils and Li displacements were significant. It means that in our study, vertical and horizontal chin repositioning was significant in comparison to previous studies. However, horizontal superior lip sulcus changes showed to be significant either in the present study. Furthermore, since profile score of most patients was above 3 (moderate), horizontal displacement of anterior segment of the maxilla (i.e. point A) seemed to be necessary obtaining the best profile view. 

Investigating the correlation between hard and soft tissue displacements, Shiu-Shiung lin performed a study in vertical and horizontal dimensions (double-jaw surgery), and the average (B and Ils) and (Pog and Pog’) demonstrated the highest correlations.^11^ In two systematic reviews by Kaklamanos *et al.*^12^ and Olate *et al.*^13^ and a study by Gjorup *et al.* (mandibular setback surgery),^14^ (B and Ils) and (Pog and Pog’) showed the highest correlation. Besides, the results in a study by Altung Atac *et al.* (double-jaw surgery) and (B and ILs) showed the highest correlation (horizontal dimension).^10^ In the present study, (Pog and Pog’), (B and Ils) and (A and Sls) in horizontal and vertical dimensions were highly correlated, and (Pog and Pog’) coming next (B and Ils) illustrated the greatest correlation in comparison to the previous studies, and in all three regions, the correlation of horizontal displacements were more than the vertical ones.

Likewise, in a study by Marsan *et al.* (double-jaw Surgery),^15^ the upper lip length of the patients generally increased. In study by Altung Atac (double-jaw surgery)^10^ and Chunmaneechote (mandibular setback surgery),^16^ the upper lip thickness decreased. However, in the present study, the upper lip thickness decreased in 14 cases and increased in 11 subjects. Further investigation is needed to understand and document the manipulations performed by each surgeon on the anterior part of nasomaxillary complex. In this study, like Chunmaneechote’s study (mandibular setback surgery),^16^ soft tissue thickness did not show any strong evidence as a controller of soft and hard tissue correlation. Unlike the study by Gjorup *et al.* (mandibular setback surgery), soft tissue displacement in the three upper lip, lower lip, and chin areas have been affected by the soft tissue thickness.^14^

## CONCLUSION

The results of this study in double-jaw surgery for Class III skeletal patients showed that in class III skeletal surgery, anterior displacement of point A and superior position of point B (decreasing anterior facial height) and decreasing mandibular plane angle and increasing nasolabial angle are necessary to obtain best result. The chin prominence area illustrated the highest predictability of the soft tissue displacement. This predictability is more in horizontal dimension than the vertical one. Upper lip length increases in most of Class III patients after orthognathic surgery. Soft tissue thickness had no effect on predicting soft tissue over the hard tissue.

## CONFLICT OF INTEREST

The authors declare no conflict of interest.
